# Confirmation bias in human reinforcement learning: Evidence from counterfactual feedback processing

**DOI:** 10.1371/journal.pcbi.1005684

**Published:** 2017-08-11

**Authors:** Stefano Palminteri, Germain Lefebvre, Emma J. Kilford, Sarah-Jayne Blakemore

**Affiliations:** 1 Institute of Cognitive Neuroscience, University College London, London, United Kingdom; 2 Laboratoire de Neurosciences Cognitives, Institut National de la Santé et de la Recherche Médicale, Paris, FR; 3 Departement d’Études Cognitives, École Normale Supérieure, Paris, FR; 4 Institut d’Études de la Cognition, Université de Recherche Paris Sciences et Lettres, Paris, FR; 5 Laboratoire d’Économie Mathématique et de Microéconomie Appliquée, Université Panthéon-Assas, Paris, FR; Johns Hopkins University, UNITED STATES

## Abstract

Previous studies suggest that *factual* learning, that is, learning from obtained outcomes, is biased, such that participants preferentially take into account positive, as compared to negative, prediction errors. However, whether or not the prediction error valence also affects *counterfactual* learning, that is, learning from forgone outcomes, is unknown. To address this question, we analysed the performance of two groups of participants on reinforcement learning tasks using a computational model that was adapted to test if prediction error valence influences learning. We carried out two experiments: in the factual learning experiment, participants learned from partial feedback (i.e., the outcome of the chosen option only); in the counterfactual learning experiment, participants learned from complete feedback information (i.e., the outcomes of both the chosen and unchosen option were displayed). In the factual learning experiment, we replicated previous findings of a valence-induced bias, whereby participants learned preferentially from positive, relative to negative, prediction errors. In contrast, for counterfactual learning, we found the opposite valence-induced bias: negative prediction errors were preferentially taken into account, relative to positive ones. When considering valence-induced bias in the context of both factual and counterfactual learning, it appears that people tend to preferentially take into account information that confirms their current choice.

## Author summary

While the investigation of decision-making biases has a long history in economics and psychology, learning biases have been much less systematically investigated. This is surprising as most of the choices we deal with in everyday life are recurrent, thus allowing learning to occur and therefore influencing future decision-making. Combining behavioural testing and computational modeling, here we show that the valence of an outcome biases both factual and counterfactual learning. When considering factual and counterfactual learning together, it appears that people tend to preferentially take into account information that confirms their current choice. Increasing our understanding of learning biases will enable the refinement of existing models of value-based decision-making.

## Introduction

Goal-directed behaviour is composed of two core components [1]: one component is the decision-making process that starts with representing the available options and terminates in selecting the option with the highest expected value; the second component is reinforcement learning (RL), through which outcomes are used to refine value expectations, in order to improve decision-making. Human decision-making is subject to biases (i.e. deviations from the normative prescriptions), such as the framing effect [2]. While the investigation of decision-making biases has a long history in economics and psychology, learning biases have been much less systematically investigated [3]. This is surprising as most of the decisions we deal with in everyday life are experience-based and choice contexts are recurrent, thus allowing learning to occur and therefore influencing future decision-making. In addition, it is important to investigate learning biases as there is evidence that RL processes play a role in psychiatric conditions and maladaptive economic behaviour [4,5].

Standard RL algorithms learn action-outcome associations directly from obtained outcomes on a trial and error basis [6]. We refer to this direct form of learning as “factual learning”. Despite the fact that standard models, built around the notion of computational and statistical optimality, prescribe that an agent should learn equally well from positive and negative obtained outcomes [7–9], previous studies have consistently shown that humans display a significant valence-induced bias. This bias generally goes in the direction of preferential learning from positive, compared to negative, outcome prediction errors [10–14]. This learning asymmetry could represent a RL counterpart of the “good news/bad news” effect that is observed for probabilistic reasoning [15].

However, human RL cannot be reduced simply to learning from obtained outcomes. Other sources of information can be successfully integrated in order to improve performance and RL has a multi-modular structure [16]. Amongst the more sophisticated learning processes that have already been demonstrated in humans is counterfactual learning. Counterfactual learning refers to the ability to learn from forgone outcomes (i.e. the outcomes of the option(s) that were not chosen) [17,18]. Whether or not a valence-induced bias also affects counterfactual learning remains unknown.

**Fig 1:**
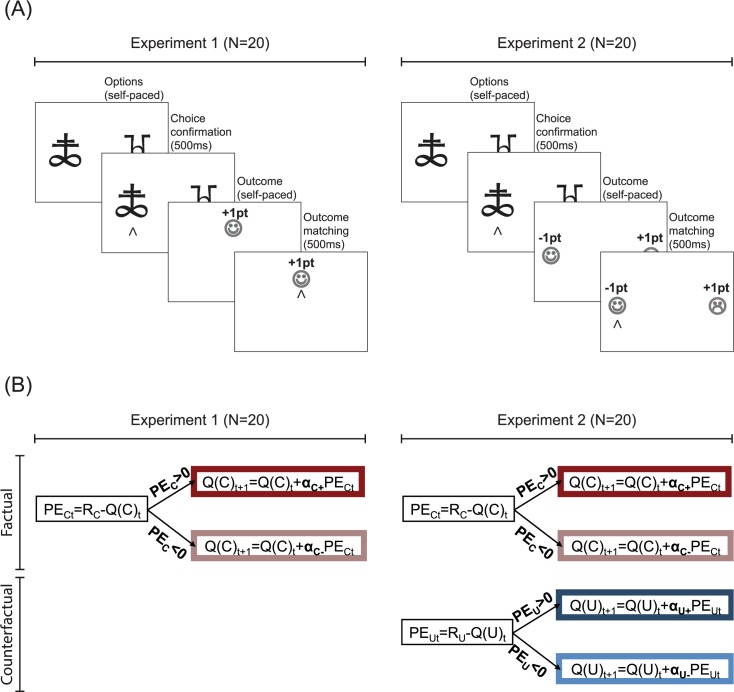
Behavioural task variants and computational model. (A) Behavioural task variants. In Experiment 1 (leftmost panel) participants were shown only the outcome of the chosen option. In Experiment 2 (rightmost panel) participants were shown the outcome of both the chosen and the unchosen options. (B) Computational models. The schematic summarises the value update stage of our computational model. The model contains two computational modules, a factual learning module (in red) to learn from chosen outcomes (R_C_) and a counterfactual learning module (in blue) to learn from unchosen outcomes (R_U_) (note that the counterfactual learning module does not apply to Experiment 1). Chosen (Q_C_) and unchosen (Q_U_) option values are updated with delta rules that use different learning rates for positive and negative factual (PE_C_) and counterfactual prediction errors (PE_U_).

To address this question, we ran two experiments involving instrumental learning and computational model-based analyses. Two groups of healthy adults performed variants of a repeated two-armed bandit task involving probabilistic outcomes [19,20] (**Fig 1A**). We analysed the data using a modified Rescorla-Wagner model that assumes different learning rates for positive and negative, and factual and counterfactual, prediction errors (**Fig 1B**) [21,22].

**Fig 2:**
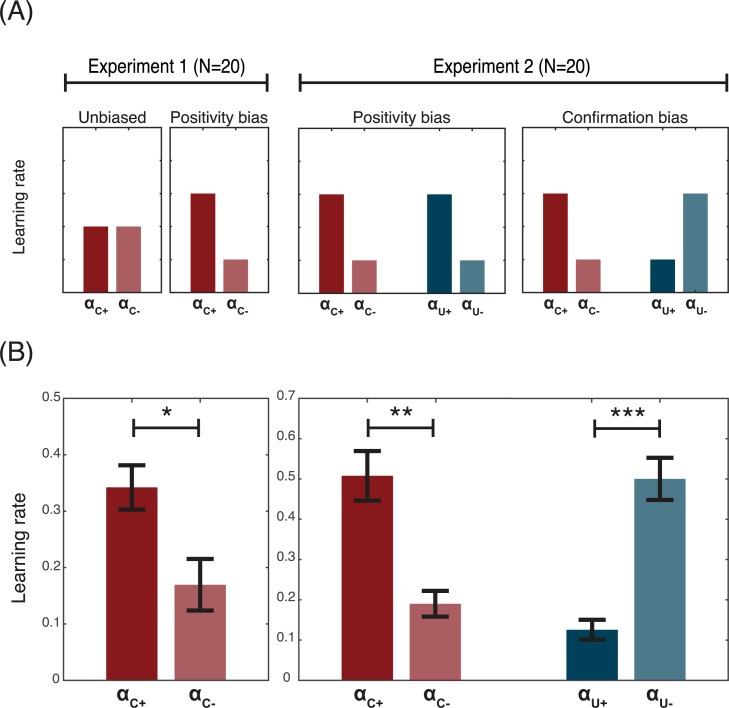
Factual and counterfactual learning biases. (A) Predicted results. Based on previous studies we expected that in Experiment 1 factual learning would display a “positivity” bias (i.e. the learning rate for the chosen positive outcomes would be relatively higher than that of the chosen negative outcomes ($\alpha_{c}^{+}>\alpha_{c}^{-}$; note that in Experiment 1 the “positivity” and the “confirmation” bias are not discernible). In Experiment 2, one possibility was that this “positivity” bias would extend to counterfactual learning, whereby positive outcomes would be over-weighted regardless of whether the outcome was chosen or unchosen (“valence” bias) ($\alpha_{u}^{+}>\alpha_{u}^{-}$). Another possibility was that counterfactual learning would present an opposite bias, whereby the learning rate for unchosen negative outcomes was higher than the learning rate of unchosen positive outcomes ($\alpha_{u}^{+}<\alpha_{u}^{-}$) (“confirmation” bias). (B) Actual results. Learning rate analysis of Experiment 1 data replicated previous findings, demonstrating that factual learning presents a “positivity” bias. Learning rate analysis of Experiment 2 indicated that counterfactual learning was also biased, in a direction that was consistent with a “confirmation” bias. ***P<0.001 and *P<0.05, two-tailed paired t-test.

The first experiment aimed to replicate previous findings of a “positivity bias” at the level of factual learning. In this first experiment, participants were presented only with the obtained outcome (chosen outcome: R_C_; **Fig 1A**) [10]. In the second experiment, in order to investigate whether or not counterfactual learning rates are also affected by the valence of prediction errors, we used a variant of the same instrumental learning task, in which participants were also presented with the forgone outcome (unchosen outcome: R_U_; **Fig 1B**). Our design allowed us to test three competing hypotheses concerning the effect of valence on counterfactual learning (**Fig 2A**). The first hypothesis–“no bias”—was that unlike factual learning, counterfactual learning would be unbiased. The second hypothesis,—“positivity bias”—was that factual and counterfactual learning would present the same valence-induced bias, such that positive counterfactual prediction errors would be more likely to be taken into account than negative counterfactual prediction errors. In this scenario, factual and counterfactual learning biases would be consequences of a more general positivity bias, in which positive prediction errors have a greater impact on learning, regardless of whether the option was chosen or not. Finally, the third hypothesis–“confirmation bias”—was that valence would affect factual and counterfactual learning in opposing directions, such that negative unchosen prediction errors would be more likely to be taken into account than positive unchosen prediction errors. In this scenario, factual and counterfactual learning biases would be consequences of a more general confirmation bias, in which outcomes that support the current choice are preferentially taken into account.

## Results

### Behavioural task and full computational model

To investigate both factual and counterfactual reinforcement learning biases, we designed an instrumental task based on a previous paradigm, in which we showed a significant positivity bias in factual learning [10]. Here, we used two variants of the task, which differed in that the task used in Experiment 1 involved participants (N = 20) being shown only the outcome of their chosen option, whereas in Experiment 2 (N = 20) the outcome of the unchosen option was also displayed (**Fig 1A**). To test our hypotheses concerning valence-induced learning biases (**Fig 2A**) we fitted the data with a Rescorla-Wagner model assuming different learning rates for positive and negative outcomes, which respectively elicit positive and negative prediction errors (**Fig 1B**). The algorithm used to explain Experiment 1 data involved two learning rates for obtained outcomes ($\alpha_{c}^{+}$ and $\alpha_{c}^{-}$ for positive and negative prediction errors of the obtained outcomes, respectively). In addition to the obtained outcome learning rates, the algorithm used to explain Experiment 2 data also involved two learning rates for forgone outcomes ($\alpha_{u}^{+}$ and $\alpha_{u}^{-}$ for positive and negative prediction errors of the forgone outcomes, respectively).

### Learning rate analysis

Replicating previous findings, in Experiment 1 we found that the positive factual learning rate ($\alpha_{c}^{+}$) was significantly higher than the negative one ($\alpha_{c}^{-}$; T(19) = 2.4; P = 0.03) (**Fig 2B**, left). In Experiment 2, we analysed learning rates using a repeated-measure ANOVA with prediction error valence (positive or negative) and prediction error type (factual or counterfactual) as within-subjects factors. Falsifying the “positivity bias” hypothesis, the ANOVA revealed no main effect of prediction error valence (F(1,19) = 0.2; P>0.6). We also did not find any effect of prediction error type, indicating that, on average, factual and counterfactual learning were similar (F(1,19) = 0.5; P>0.4). Consistent with the “confirmation bias” hypothesis, we found a significant interaction between valence and type (F(1,19) = 119.2; P = 1.3e-9). Post-hoc tests indicated that the interaction was driven by effects of valence on both factual ($\alpha_{c}^{+}>\alpha_{c}^{-}$; T(19) = 3.6; P = 0.0017) and counterfactual learning rates ($\alpha_{u}^{-}>\alpha_{u}^{+}$; T(19) = 6.2; P = 5.8e-06) (**Fig 2B,** right).

To verify the robustness of this result in the context of different reward contingencies, we analysed learning rates in each task condition separately. In both experiments, our task included three different conditions (**S1 Fig**): a “Symmetric” condition, in which both options were associated with a 50% chance of getting a reward; an “Asymmetric” condition, in which one option was associated with a 75% chance of getting a reward, whereas the other option was associated with only a 25% chance; and a “Reversal” condition, in which one option was initially associated with a 83% chance of getting a reward and the other option was associated with a 17% chance of getting a reward, but after 12 trials the reward contingencies were reversed. For Experiment 1, we analysed factual learning rates using a repeated-measure ANOVA with prediction error valence (positive and negative) and task condition (Symmetric, Asymmetric and Reversal) as within-subjects factors (**S1B Fig**). Confirming the aggregate result, the ANOVA showed a significant main effect of valence (F(1,19) = 26.4, P = 5.8e-5), but no effect of condition (F(2,38) = 0.7, P>0.5), and, crucially, no valence by condition interaction (F(2,38) = 0.8, P>0.4). For Experiment 2, we analysed factual and counterfactual learning rates using a repeated-measure ANOVA with prediction error valence (positive and negative), prediction error type (factual or counterfactual) and condition (Symmetric, Asymmetric and Reversal) as within-subjects factors (**S1C Fig**). Confirming the aggregate result, the ANOVA showed no effect of prediction error type (F(1,19) = 0.0, P>0.9), no effect of valence (F(1,19) = 0.3, P>0.5), but a significant valence by type interaction (F(1,19) = 162.9, P = 9.1e-11). We also found an effect of condition (F(2,38) = 5.1, P = 0.01), reflecting lower average learning rates in the Reversal compared to the Asymmetric condition (T(19) = 2.99; P = 0.007), which was not modulated by valence (F(2,38) = 0.2, P>0.7), or type (F(2,38) = 1.2, P>0.3). The three-way interaction was not significant (F(2,38) = 1.8, P>.1), indicating that learning biases were robust across different task contingencies.

### Dimensionality reduction with model comparison

**Fig 3:**
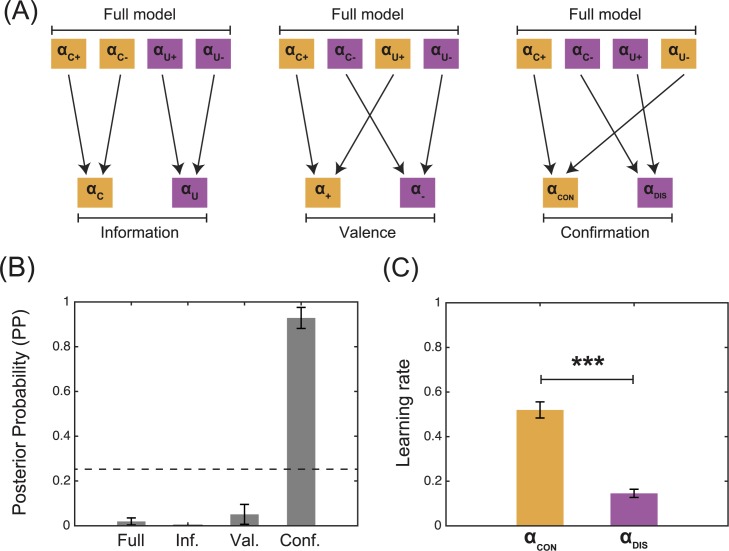
Dimensionality reduction with model comparison. (A) Model space. The figure represents how the number of parameters (learning rates) are reduced moving from the ‘Full’ model to more simple ones. (B) Model comparison. The panel represents the posterior probability (PP) of the models, the calculation of which is based on the BIC, which penalisses model complexity. The dashed line represents random posterior probability (0.25). (C) Model parameters. The panel represents the learning rate for the best fitting model (i.e., the ‘Confirmation’) model. α_CON_: learning rate for positive obtained and negative forgone outcomes; α_DIS_: learning rate for negative obtained and positive forgone outcomes. ***P<0.001, two-tailed paired t-test.

To further test our hypotheses and verify theparsimony of our findings, we ran a model comparison analysis including the ‘Full’ model (i.e., the model with four learning rates; **Fig 1C,** right) and reduced, alternative versions of it (**Fig 3A**). The first alternative model was obtained by reducing the number of learning rates along the dimension of the outcome type (factual or counterfactual). This ‘Information’ model has only two learning rates: one for the obtained outcomes (α_C_) and another for the forgone outcomes (α_U_). The second alternative model was obtained by reducing the number of learning rates along the dimension of the outcome valence (positive or negative). This ‘Valence’ model has only two learning rates (one for the positive outcomes (α_+_) and another for the negative outcomes (α_-_)) and should win according to the “positivity bias” hypothesis. Finally, the third alternative model was obtained by reducing the learning rate as a function of the outcome event being confirmatory (positive obtained or negative forgone) or disconfirmatory (negative obtained or positive forgone). This ‘Confirmation’ model has only two learning rates (one for confirmatory outcomes (α_CON_) and another for the disconfirmatory outcomes (α_DIS_)) and should win according to the “confirmation bias” hypothesis.

Bayesian Information Criterion (BIC) analysis indicated that the ‘Full’ model better accounted for the data compared to both the ‘Information’ and the ‘Valence’ models (both comparisons: T(19)>4.2; P<0.0005; **Table 1**). However the ‘Confirmation’ model better accounted for the data compared to the ‘Full’ model (T(19) = 9.9; P = 6.4e-9). The posterior probability (PP) of belonging to each model, calculated for each subject, (i.e., the averaged individual model attributions) of the ‘Confirmation’ model was higher than chance (.0.25 for a model space including 4 models; T(19) = 13.5; P = 3.3e-11) and higher than the posterior probability all the other models (all comparisons: T(19)>9.0; P<2.1e-8) (**Fig 3B**). The learning rate for confirmatory outcomes was significantly higher than that for disconfirmatory outcomes (α_CON_>α_DIS_; T(19) = 11.7; P = 3.9e-10) (**Fig 3C**). These results support the “confirmation bias” hypothesis and further indicate that, at least at the behavioural level, chosen and unchosen outcomes may be processed by the same learning systems.

**Table 1:** Model comparison. The “winning” model is the “Confirmation” model for which the learning rates are displayed in **Fig 3C**. The second best model is the Full model, for which the learning rates are displayed in **Fig 2B**.

Model	Full (5df)	Information (3df)	Valence (3df)	Confirmation (3df)	Perseveration (4df)	One (2df)
**BIC**	162.0±13.4	178.2±13.0	180.7±11.8	155.0±13.2	165.2±13.6	179.1±11.8
**PP**	0.02±0.02	0.00±0.00	0.05±0.05	0.89±0.06	0.01±0.01	0.04±0.03
**XP**	0.00	0.0	0.0	1.0	0.0	0.0

BIC: Bayesian Information Criterion; PP: posterior probability; XP: exceedance probability; df: degrees of freedom.

### Comparison between the learning curves and the model estimates

**Fig 4:**
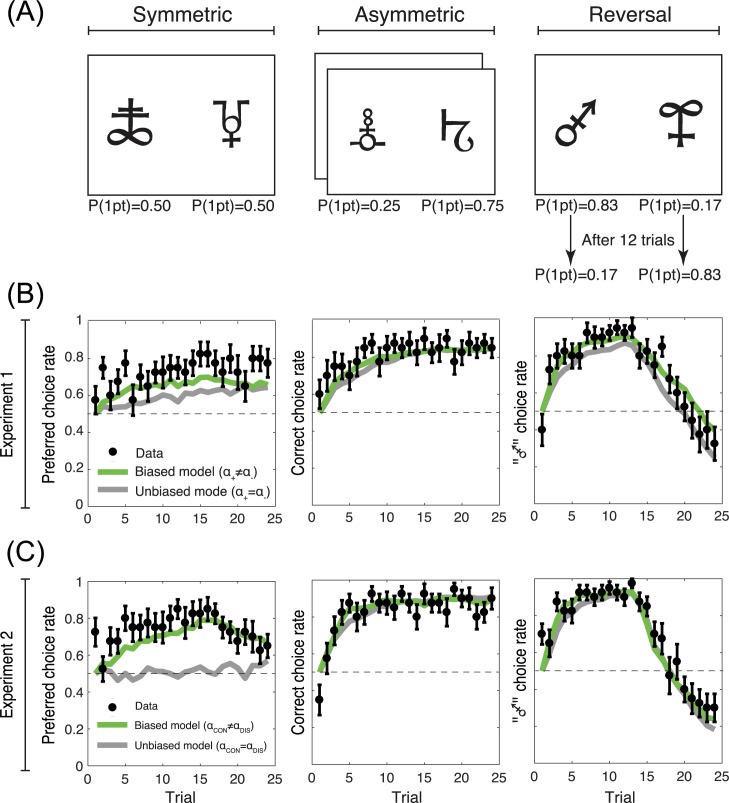
Learning curves and model estimates. (A) Task conditions. (B) and (C) Learning curves as a function of the task conditions in Experiment 1 and Experiment 2, respectively. Each panel displays the result of the corresponding condition presented in (A). The black dots and the error bars represent the actual data ± s.e.m. The green lines represent the model estimates of the biased models (Experiment 1: $\alpha_{c}^{+}\neq \alpha_{c}^{-}$; Experiment 2:α_CON_≠α_DIS_), the grey lines represent the model estimates of the unbiased models (Experiment 1: $\alpha_{c}^{+}=\alpha_{c}^{-}$; Experiment 2: α_CON_ = α_DIS_).

To evaluate the capacity of our models to reproduce the learning curves, we plotted and analysed the trial-by-trial model estimates of choice probabilities (**Fig 4**) [23]. The model estimates were generated using the best fitting set of parameters for each individual and model. In the Symmetric condition (where there is no correct response), we considered the preferred option choice rate (i.e., the option/symbol that was chosen more than >50%). In the Asymmetric condition we considered the correct choice rate. In the Reversal condition (where the correct response is reversed after the first half of the trials) we considered the choice rate of the initially more advantageous option (i.e., the correct option during the first half). Qualitative observation of the learning curves indicated that the biased models (Experiment 1: $\alpha_{c}^{+}\neq \alpha_{c}^{-}$; Experiment 2: α_CON_≠α_DIS_) tended to reproduce the learning curves more closely. To quantify this, we compared the average square distance between the biased and the unbiased models (Experiment 1: $\alpha_{c}^{+}=\alpha_{c}^{-}$; Experiment 2: α_CON_ = α_DIS_). We found that the square distance was shorter for the biased models compared to the unbiased models in both experiments (Experiment 1: 0.074 vs. 0.085, T(19) = 3.5 P = 0.0022; Experiment 2: 0.056 vs. 0.064, T(19) = 3.5 P = 0.0016).

### Parameter correlation and parameter recovery

**Fig 5:**
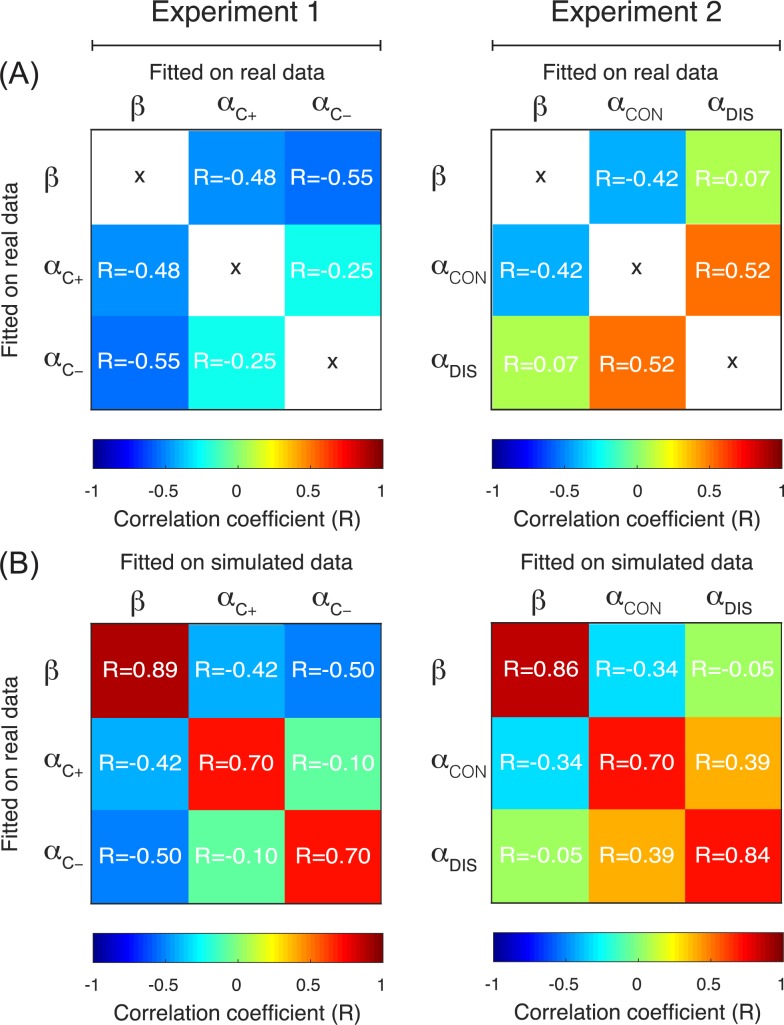
Parameter correlation and recovery. (A) Correlation matrix of the free parameters for Experiment 1 (left) and Experiment 2 (right). Dark blue or dark red values of R indicate a strong correlation and therefore a problem in parameter identifiability. (B) Correlation matrix of the free parameters used to generate the simulated data (‘Fitted on real data’) and obtained by applying the parameter estimation procedure on the simulated data (‘Fitted on simulated data’). Dark red values of R indicate a strong correlation between the true and the retrieved parameter value and therefore a good parameter recovery.

We calculated the Pearson correlation between the parameters (**Fig 5A**) and found no significant correlation when correcting for multiple comparisons (corrected P value = 0.05÷6 = 0.008; lowest uncorrected P value = 0.01, highest P^2^ = 0.30). The correlation between α_CON_ and α_DIS_ was weak, but positive, which rules out the possibility that the significant difference between these two learning rates was driven by an anti-correlation induced by the model fitting procedure.

We then applied the same model fitting procedure to the synthetic datasets and calculated the correlation between the true and the retrieved parameters (**Fig 5B**). We found that, on average, all parameters in both experiments were well recovered (0.70 ≤ R ≤ 0.89) and that our model fitting procedure introduced no spurious correlations between the other parameters (|R| ≤ 0.5).

We also checked the parameter recovery for discrete sets of parameter values (**S2**& **S3 Figs**). For Experiment 1, we simulated unbiased ($\alpha_{c}^{+}=\alpha_{c}^{-}$) and biased ($\alpha_{c}^{+}>\alpha_{c}^{-}$) participants. For Experiment 2, we simulated unbiased ($\alpha_{c}^{+}=\alpha_{c}^{-}$ and $\alpha_{u}^{+}=\alpha_{u}^{-}$), semi-biased ($\alpha_{c}^{+}>\alpha_{c}^{-}$ and $\alpha_{u}^{+}=\alpha_{u}^{-}$) and biased ($\alpha_{c}^{+}>\alpha_{c}^{-}$ and $\alpha_{u}^{+}>\alpha_{u}^{-}$) participants. We simulated N = 100 virtual participants per set of parameters. The results of these analyses are presented in the supplementary materials and confirm the capacity of our parameter optimisation procedure to correctly recover the true parameters, regardless of the presence (or absence) of learning rate biases.

### Behavioural signatures of learning biases

To investigate the behavioural consequences of the learning biases, we median-split the participants from each experiment into two groups according to their normalised learning rate differences. We reasoned that the effects of learning biases on behavioural performance could be highlighted by comparing participants who differed in the extent they expressed the bias itself. Experiment 1 participants were split according to their normalised factual learning rate bias: $\left(\alpha_{c}^{+}-\alpha_{c}^{-})/(\alpha_{c}^{+}+\alpha_{c}^{-}\right)$, from which we obtained a high (M = 0.76±0.05) and a low bias (M = 0.11±0.14) group. Experiment 2 participants were split according their normalised confirmation bias: $\left[(\alpha_{c}^{+}-\alpha_{c}^{-})-(\alpha_{u}^{+}+\alpha_{u}^{-})]/(\alpha_{c}^{+}+\alpha_{c}^{-}+\alpha_{u}^{+}+\alpha_{u}^{-}\right)$, from which we also obtained a high bias group (M = 0.72±0.04) and a low bias group (M = 0.36±0.04).

**Fig 6:**
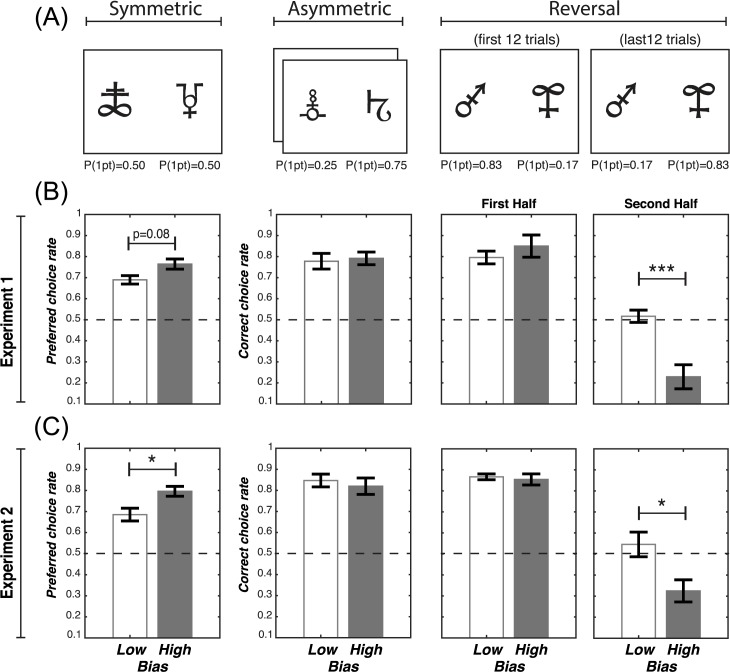
Behavioural signatures distinguishing “low” and “high” bias participants. (A) Task conditions. The ‘Symmetric’ condition was characterised by a stable reward contingency and no correct option, because the two options had equal reward probabilities. The ‘Asymmetric conditions’ were also characterised by a stable reward contingency but had a correct option, since one option had a higher reward probability than the other. The ‘Reversal’ condition was characterised by an unstable reward contingency: after 12 trials the reward probability reversed across symbols, so that the former correct option became the incorrect one, and vice versa. Note that the number of trials refers to one session and participants performed two sessions, each involving new pairs of stimuli (192 trials in total). (B) and (C) Behavioural results as a function of the task conditions in Experiment 1 and Experiment 2, respectively. Each column presents the result of the corresponding condition presented in (A). In the Symmetric condition, where there was no correct option, we calculated the “preferred choice rate”, which was the choice rate of the most frequently chosen option (by definition, this was always greater than 0.5). In the Asymmetric and the Reversal conditions we calculated the correct choice rate. In the Reversal condition the correct choice rate was split between the two learning phases. ***P<0.001 and *P<0.05, two-tailed paired t-test.

From the Symmetric condition we extracted preferred choice rate as a dependent variable, which was the choice rate of the most frequently chosen option (i.e. the option that was chosen on >50% of trials) (**Fig 6A**). We hypothesised that higher biases were associated with an increased tendency to develop a preferred choice, even in the absence of a “correct” option, which naturally emerges from overweighting positive factual (and/or negative counterfactual) outcomes, as observed in our previous study [10]. We submitted the preferred choice rate to an ANOVA with experiment (1 vs. 2) and bias level (high vs. low) as between-subjects factors. The ANOVA showed a significant main effect of bias level (F(1,36) = 8.8, P = 0.006). There was no significant main effect of experiment (F(1,36) = 0.6, P>0.6) and no significant interaction between experiment and bias level (F(1,36) = 0.3, P>0.5). Replicating previous findings, the main effect of bias level was driven by higher preferred choice rate in the high, compared to the low bias group in both Experiment 1 (T(18) = 1.8 P = 0.08) and Experiment 2 (T(18) = 2.3 P = 0.03) (**Fig 6B & 6C**).

From the remaining conditions we extracted the correct choice rate, which was the choice rate of the most frequently rewarded option. In the Reversal condition, correct choice rate was split across the first half of the trial (i.e., before the reversal of the contingencies) and second half (i.e., after the reversal of the contingencies) (**Fig 6A**). We hypothesised that in the second half of the Reversal condition, where correct choice rate depends on un-learning previous associations based on negative factual prediction errors (and positive counterfactual prediction errors, in Experiment 2), high bias subjects will display reduced performance. We submitted the correct choice rate to a mixed ANOVA with experiment (1 vs. 2) and bias group (high vs. low) as between-subjects factors, and condition (Asymmetric, Reversal: first half, and Reversal: second half) as a within-subjects factor. There was a main effect of experiment (F(1,36) = 4.1, P = 0.05), indicating that correct choice rate was higher in Experiment 2 than Experiment 1, which is consistent with previous studies showing that counterfactual feedback enhances learning[20,24]. We also found a significant effect of bias level (F(1,36) = 10.8, P = 0.002), a significant effect of condition (F(2,72) = 99.5, P = 2.0e-16), and a significant bias level by condition interaction (F(2,72) = 9.6, P = 0.0002). Indeed, in both experiments, the correct choice rate in the second half of the Reversal condition was lower in the high bias compared to the low bias group (Experiment 1: T(18) = 3.9 P = 0.0003; Experiment 2: T(18) = 2.5 P = 0.02) (**Fig 6B & 6C**).

Importantly, we found that the temperature did not differ between low and high bias subjects in Experiment 1 (low vs. high: 3.38±0.82 vs. 3.78±0.67; T(18) = 0.4, P = 0.7078) or in Experiment 2 (low vs. high. 3.29±0.56 vs. 2.13±0.36; T(18) = 1.7, P = 0.0973). Of note, the difference in temperature goes in two different directions in the two experiments, whereas the behavioural effects (i.e., increased preferred response rate in the Symmetric condition and decreased performance in the second half of the Reversal condition) go in the same direction. Finally, we used Pearson’s correlations to verify that the relevant results remained significant when assessed as continuous variables. As predicted, the normalised learning biases were significantly and positively correlated with the preferred choice rate in the Symmetric condition in both experiments (Experiment 1: R = 0.54, P = 0.013; Experiment 2: R = 0.46, P = 0.040). Similarly, the normalised learning biases were significantly and negatively correlated with the correct choice rate in the second half of the Reversal condition (Experiment 1: R = -0.66, P = 0.0015; Experiment 2: R = -0.47, P = 0.033).

## Discussion

Two groups of healthy adult participants performed two variants of an instrumental learning task, involving factual (Experiment 1) and counterfactual (Experiments 1 & 2) reinforcement learning. We found that prediction error valence biased factual and counterfactual learning in opposite directions. Replicating previous findings, we found that, when learning from obtained outcomes (factual learning), the learning rate for positive prediction errors was higher than the learning rate for negative prediction errors. In contrast, when learning from forgone outcomes (counterfactual learning), the learning rate for positive prediction errors was lower than that of negative prediction errors. This result proved stable across different reward contingency conditions and was further supported by model comparison analyses, which indicated that the most parsimonious model was a model with different learning rates for confirmatory and disconfirmatory events, regardless of outcome type (factual or counterfactual) and valence (positive or negative). Finally, behavioural analyses showed that participants with a higher valence-induced learning bias displayed poorer learning performance, specifically when it was necessary to adjust their behaviour in response to a reversal of reward contingencies. These learning biases were therefore significantly associated with reduced learning performance and can be considered maladaptive in the context or our experimental tasks.

Our results demonstrated a factual learning bias, which replicates previous findings by showing that, in simple instrumental learning tasks, participants preferentially learn from positive compared to negative prediction errors [11–13]. However, in contrast to previous studies, in which this learning bias had no negative impact on behavioural performance (i.e., correct choice rate and therefore final payoff), here we demonstrated that this learning bias is still present in situations in which it has a negative impact on performance. In fact, whereas low and high bias participants performed equally well in conditions with stable reward contingencies, in conditions with unstable reward contingencies we found that high bias participants showed a relatively reduced correct choice rate. When reward contingencies were changed, learning to successfully reverse the response in the second half of the trials was mainly driven by negative factual (and positive counterfactual) prediction errors. Thus in this case, participants displaying higher biases exhibited a lower correct choice rate. In other words, these learning biases significantly undermined participants’ capacity to flexibly adapt their behaviour in changing, uncertain environments.

In addition to reduced reversal learning, and in accordance with a previous study [10], another behavioural feature that distinguished higher and lower bias participants was the preferred response rate in the Symmetric condition. In the Symmetric condition, both cues had the same reward probabilities (50%), such that there was no intrinsic “correct” response. This allowed us to calculate the preferred response rate for each participant (defined as the choice rate of the option most frequently selected by a given participant, i.e. the option selected in > 50% of trials). The preferred response rate can therefore be taken as a measure of the tendency to overestimate the value of one cue compared to the other, in the absence of actual outcome-based evidence. In both experiments, higher bias participants showed higher preferred response rates, a behavioural pattern that is consistent with an increased tendency to discount negative factual (and positive counterfactual) prediction errors. This can result in one considering a previously rewarded chosen option as better than it really is and an increased preference for this choice. Thus, these results illustrate that the higher the learning bias for a given participant, the higher his/her behavioural perseveration (the tendency to repeat a previous choice), despite the possible acquisition of new evidence in the form of negative feedback.

Previous studies have been unable to distinguish whether this valence-induced factual learning bias is a “positivity bias” or a “confirmation bias”. In other words, do participants preferentially learn from positive prediction errors because they are positively valenced or because the outcome confirms the choice they have just made? To address this question we designed Experiment 2 in which, by including counterfactual feedback, we were able to separate the influence of prediction error valence (positive vs. negative) from the influence of prediction error type (chosen vs. unchosen outcome). Crucially, whereas the two competing hypotheses (“positivity bias” vs. “confirmation bias”) predicted the same result concerning factual learning rates, they predicted opposite effects of valence on counterfactual learning rates. The results from Experiment 2 support the confirmation bias hypothesis: participants preferentially took into account the outcomes that confirmed their current behavioural policy (positive chosen and negative unchosen outcomes) and discounted the outcomes that contradicted it (negative chosen and positive unchosen outcomes). Our results therefore support the idea that confirmation biases are pervasive in human cognition [25].

It should be noted that, from an orthodox Bayesian perspective, a confirmation bias would involve reinforcing one's own initial beliefs or preferences. Previous studies have investigated how prior information—in the form of explicit task instructions or advice—influences the learning of reinforcement statistics and have provided evidence of a confirmation bias [26–28]. However, consistent with our study, their computational and neural results suggest that this instruction-induced confirmation bias operates at the level of outcome processing and not at the level of initial preferences or at the level of the decision-making process [29,30]. Here, we take a slightly different perspective by extending the notion of confirmation bias to the implicit reinforcement of one's own current choice, by preferentially learning from desirable outcomes, independently from explicit prior information.

We performed a learning rate analysis separately for each task condition and the results proved robust and were not driven by any particular reward contingency condition. Our results contrast with previous studies that have found learning rates adapt as a function of task contingencies, showing increases when task contingencies were unstable [31,32]. Several differences between these tasks and ours may explain this discrepancy. First, in previous studies, the stable and unstable phases were clearly separated, whereas in our design, participants were simultaneously tested in the three reward contingency conditions. Second, we did not explicitly tell participants to monitor the stability of the reward contingency. Finally, since in our task the Reversal condition represented only one quarter of the trials, participants may not have explicitly realised that changing learning rates were adaptive in some cases.

To date, two different views of counterfactual learning have been proposed. According to one view, factual and counterfactual learning are underpinned by different systems that could be computationally and anatomically mapped onto subcortical, model-free modules, and prefrontal, model-based modules [17,18,33]. In contrast, according to another view, factual and counterfactual outcomes are processed by the same learning system, involving the dopaminergic nuclei and their projections [34–36]. Our dimensionality reduction model comparison result sheds new light on this debate. If the first view was correct, and factual and counterfactual learning are based on different systems, different learning rates for positive and negative prediction errors would have better accounted for the data (the ‘Information’ model). In contrast, our results showed that the winning model was one in which the learning process was assumed to be different across desirable and undesirable outcomes, but shared across obtained and forgone outcomes (as in the “Confirmation” model), This supports the second view that factual and counterfactual learning are different facets of the same system.

Overall, we found that correct choice rate was higher in Experiment 2 than in Experiment 1, indicating that the presence of complete feedback information improved performance. Previous literature in psychology and economics suggest that this beneficial effect of counterfactual information is conditional on the payoff structure of the task. Specifically, studies have shown that the presence of rare positive outcomes could impair performance in the presence of complete feedback [37–40]. Further research is needed to assess whether or not the learning biases we identified extend to these payoff schemes and how they relate to the observed performance impairment.

Another series of studies in psychology and economics have used paradigms that dissociate information sampling (i.e., choosing an option to discover its value without getting the outcome) from actual choice (i.e., choosing an option in order to obtain the associated outcome) [3]. Other paradigms have been used to investigate learning from outcomes derived from choices performed by either a computer or another player (i.e., observational learning) [41,42]. Future research should assess whether or not information sampling and observational learning present similar valence-induced learning biases.

Why do these learning biases exist? One possibility is that these learning biases arise from neurobiological constraints, which limit human learning capacity. However, we believe this interpretation is unlikely because we see no clear reason why such limits would differentially affect learning from positive and negative prediction errors. In other words, we would predict that neurobiological constraints on learning rate would limit all learning rates in a similar way and therefore not produce valence-induced learning asymmetries. A second possibility is that these learning biases are not maladaptive. For instance, it has been shown that in certain reward conditions agents displaying valence-induced learning biases may outperform unbiased agents [9]. Thus, a possible explanation for these learning biases is that they have been positively selected because they can be adaptive in the context of the natural environment in which the learning system evolved [43]. A third, intermediate possibility is that these learning biases can be maladaptive in the context of learning performance, but due to their adaptive effects in other domains of cognition, overall they have a net adaptive value. For example, these biases may also manifest as “self-serving”, choice-supportive biases, which result in individuals tending to ascribe success to their own abilities and efforts, but relatively tending to neglect their own failures [44]. Accordingly, we could speculate that these learning biases may help promote self-esteem and confidence, both of which have been associated with overall favourable real life outcomes [45].

In summary, by investigating both factual and counterfactual learning, the current experiments demonstrate that, when presented with new evidence, people tend to discard information that suggests they have made a mistake. This selective neglect of useful information may have adaptive value, by increasing self-confidence and self-esteem. However, this low level reinforcement-learning bias may represent a computational building block for higher level cognitive biases such as belief perseverance, that is, the phenomenon that beliefs are remarkably resilient in the face of empirical challenges that logically contradict them [46,47].

## Methods

### Participants

The study included two experiments. Each experiment involved N = 20 participants (Experiment 1: 7 males, mean age 23.9 ± 0.7; Experiment 2: 4 males, mean age 22.8 ± 0.7). The local ethics committee approved the study. All participants gave written informed consent before inclusion in the study, which was carried out in accordance with the declaration of Helsinki (1964, revised 2013). The inclusion criteria were being older than 18 years and reporting no history of neurological or psychiatric disorders.

### Behavioural tasks

Participants performed a probabilistic instrumental learning task based on previous studies [19,20] (**Fig 1A**). Briefly, the task involved choosing between two cues that were presented in fixed pairs and therefore represented fixed choice contexts. Cues were associated with stationary outcome probabilities in three out of four contexts. In the remaining context the outcome probability was non-stationary. The possible outcomes were either winning or losing a point. To allow learning, each context was presented in 24 trials. Each session comprised the four learning contexts and therefore included 96 trials. The whole experiment involved two sessions, each including the same number of contexts and conditions, but a different set of stimuli. Thus, the total experiment included 192 trials. The four learning contexts (i.e. fixed pairs of cues) were divided in three conditions (**S1 Fig**). In the “Symmetric” condition each cue was associated with a .50 probability of winning one point. In the “Asymmetric” condition one cue was associated with a .75 probability of winning a point and the other cue was associated with a .25 probability of winning a point. The Asymmetric condition was implemented in two choice contexts in each session. Finally, in the “Reversal” condition one cue was associated with a .83 probability of winning a point and the other cue was associated with a .17 probability of winning a point during the first 12 trials, and these contingencies were reversed thereafter. We chose a bigger probability difference in the Reversal compared to the Asymmetric condition in order to ensure that participants were able to reach a plateau within the first 12 trials. Participants were encouraged to accumulate as many points as possible and were informed that some cues would result in winning more often than others. Participants were given no explicit information regarding reward probabilities, which they had to learn through trial and error.

At each trial, after a fixation cross, the choice context was presented. Participants made their choice by pressing left or right arrow keys with their right hand (the choice time was self-paced). The two experiments differed in the fact that in the Experiment 1 participants were only informed about the outcome of their own choice (chosen outcome), whereas in the Experiment 2 participants were informed about both the obtained and the forgone outcome (i.e. counterfactual feedback). In Experiment 1 positive outcomes were presented at the top and negative outcomes at the bottom of the screen. The participant was required to press the key corresponding to the position of the outcome on the screen (top/bottom) in order to move to the subsequent trial. In Experiment 2 the obtained outcomes were presented in the same place as the chosen cues and the forgone outcomes in the same place as the unchosen cues. To move to the subsequent trial, participants had to match the position of the outcome with a key press (right/left). Importantly for our computational analyses, outcome probabilities (although on average anti-correlated in the Asymmetric and Reversal conditions) were truly independent across cues, so that in the Symmetric condition, in a given trial, the obtained and forgone outcomes were the same in 50% of trials; in the Asymmetric condition this was the case in 37.5% of trials; finally, in the Reversal condition this was the case in 28.2% of trials.

### Behavioural variables

We extracted the correct response rate, that is, the rate of the trials in which the participants chose the most rewarding response, from the Asymmetric and the Reversal conditions. The correct response rate in the Reversal condition was calculated separately for the two phases: before (“first half”) and after (“second half”) the contingency reversal. In the Symmetric condition, we calculated the so-called “preferred” response rate. The preferred response was defined as the most frequently chosen option, i.e. that chosen by the participant on more than 50% of the trials. This quantity was therefore, by definition, greater than 0.5. To investigate the behavioural consequences of learning biases on performance, we median-split the participants from each experiment into two groups according to their normalised learning rate difference (Experiment 1: $\left(\alpha_{c}^{+}-\alpha_{c}^{-})/(\alpha_{c}^{+}+\alpha_{c}^{-}\right)$; Experiment 2: $\left[(\alpha_{c}^{+}-\alpha_{c}^{-})-(\alpha_{u}^{+}+\alpha_{u}^{-})]/(\alpha_{c}^{+}+\alpha_{c}^{-}+\alpha_{u}^{+}+\alpha_{u}^{-}\right)$), from which we obtained ‘low’ and ‘high’ bias participants[48]. The preferred response rate in the Symmetric condition was submitted to an ANOVA with experiment (1 vs. 2) and bias level (high vs. low) as between-subjects factors. The correct choice rate in the remaining conditions was submitted to an ANOVA with experiment (1 vs. 2) and bias level (high vs. low) as between-subjects factors and condition (Asymmetric, Reversal first half and Reversal second half) as within-subject factors. The effects of interest identified by the ANOVAs were also confirmed using Pearson’s correlations.

### Computational models

We fitted the data with a standard Q-learning model, including different learning rates following positive and negative prediction errors and containing two different modules (**Fig 1C**): a factual learning module to learn from chosen outcomes (R_c_) and a counterfactual learning module to learn from unchosen outcomes (R_u_) (note that counterfactual learning applies only to Experiment 2). For each pair of cues (choice context), the model estimates the expected values of the two options (Q-values). These Q-values essentially represent the expected reward obtained by choosing a particular option in a given context. In both experiments, Q-values were set at 0 before learning, corresponding to the a priori expectation of a 50% chance of winning 1 point, plus a 50% chance of losing 1 point. After every trial *t*, the value of the chosen option is updated according to the following rule (factual learning module):

$$Q_{c}(t+1)=Q_{c}(t)+\begin{array}{cc} \alpha_{c}^{+}.{PE}_{c}(t) & if\;{PE}_{c}(t)>0\\ \alpha_{c}^{-}.{PE}_{c}(t) & if\;{PE}_{c}(t)<0 \end{array}$$ (1)

In this first equation, *PE*_*c*_(*t*) is the prediction error of the chosen option, calculated as:

$${PE}_{c}(t)=R_{c}(t)-Q_{c}(t),$$ (2)

where *R*_*c*_(*t*) is the reward obtained as an outcome of choosing *c* at trial *t*. In other words, the prediction error *PE*_*c*_(*t*) is the difference between the expected outcome *Q*_*c*_(*t*) and the actual outcome *R*_*c*_(*t*).

In Experiment 2 the unchosen option value is also updated according to the following rule (counterfactual learning module):

$$Q_{u}(t+1)=Q_{u}(t)+\begin{array}{cc} \alpha_{u}^{+}.{PE}_{u}(t) & if\;{PE}_{u}(t)>0\\ \alpha_{u}^{-}.{PE}_{u}(t) & if\;{PE}_{u}(t)<0 \end{array}$$ (3)

In this second equation, *PE*_*u*_(*t*) is the prediction error of the unchosen option, calculated as:

$${PE}_{u}(t)=R_{u}(t)-Q_{u}(t),$$ (4)

where *R*_*u*_(*t*) is the reward that could have been obtained as an outcome of having chosen *u* at trial *t*. In other words, the prediction error *PE*_*u*_(*t*) is the difference between the expected outcome *Q*_*u*_(*t*) and the actual outcome *R*_*u*_(*t*) of the unchosen option.

The learning rates $\alpha_{c}^{+}$ and $\alpha_{u}^{+}$ are scaling parameters that adjust the amplitude of value changes from one trial to the next when prediction errors of chosen and unchosen options, respectively, are positive (when the actual reward *R*(*t*) is better than the expected reward *Q*(*t*)). The learning rates $\alpha_{c}^{-}$ and $\alpha_{u}^{-}$ do the same when prediction errors are negative. Thus, our model allows for the amplitude of value updates to be different following positive and negative prediction errors, and for both chosen and unchosen options. It therefore allows for the existence of valence-dependent learning biases.

Finally, the probability (or likelihood) of selecting the chosen option was estimated with a soft-max rule as follows:

$$Pc(t)=e^{\left(Q_{c}(t)*\beta \right)}/\left(e^{\left(Q_{c}(t)*\beta \right)}+e^{\left(Q_{u}(t)*\beta \right)}\right).$$ (5)

This is a standard stochastic decision rule that calculates the probability of selecting one of a set of options according to their associated values. The temperature, *β*, is another scaling parameter that adjusts the stochasticity of decision-making.

In addition to this ‘Full’ model, we also considered alternative versions with a reduced number of learning rates (**Fig 3A**): the ‘Information’ model, where $\alpha_{c}^{+}=\alpha_{c}^{-}$ and $\alpha_{u}^{+}=\alpha_{u}^{-}$; the ‘Valence’ model, where $\alpha_{c}^{+}=\alpha_{u}^{+}$ and $\alpha_{c}^{-}=\alpha_{u}^{-}$; and the ‘Confirmation’ model, where $\alpha_{c}^{+}=\alpha_{u}^{-}$ and $\alpha_{c}^{-}=\alpha_{u}^{+}$. For the model comparison, we also considered a very simple model (the ‘One’) model, with only one learning rate ($\alpha_{c}^{+}=\alpha_{c}^{-}=\alpha_{u}^{+}=\alpha_{u}^{-}$), and a ‘Perseveration’ model where an additional parameter (–Inf < π < +Inf) biases the decision-making process by increasing (positive values) or decreasing (negative values) the likelihood of repeating the same choice, regardless of the previous outcome (**Table 1**).

### Parameter optimisation and model comparison

In a first analysis, we optimised model parameters by minimising the negative log-likelihood of the data, given different parameter settings, using Matlab’s fmincon function (ranges: 0<*β*<Infinite, and 0< *α*_*n*_<1):

LL=log⁡(P(Data|Model)) (6)

Negative log-likelihoods (LL) were used to compute the Bayesian information criterion (BIC) at the individual level (random effects) for each model, as follows:

BIC=log⁡(ntrials)*df+2*LL (7)

BIC were compared between biased and unbiased models to verify that the utilisation of the biased model was justified, even accounting for its extra-complexity. As an approximation of the model evidence, individual BICs were fed into the mbb-vb-toolbox [49], a procedure that estimates the exceedance probability and the model attributions for each model within a set of models, given the data gathered from all participants. Exceedance probability (denoted XP) is the probability that a given model fits the data better than all other models in the set, i.e. has the highest XP (**Table 1**). The toolbox also allows the estimation of the individual model attributions, i.e. the posterior probability (PP), for each subject, of belonging to each model. The individual model attributions can be compared to chance (defined as 1/the total number of models), compared to each other, and can also be averaged to obtain the model frequency for the population.

In a second analysis, we optimised model parameters by minimising the logarithm of the Laplace approximation to the model evidence (or log posterior probability: LPP):

LPP=log⁡(P(Data|Model,Parameters)) (8)

Because LPP maximisation includes priors over the parameters (temperature: gamma(1.2,5); learning rates beta(1.1,1.1)) [50], it avoids degenerate parameter estimates. Therefore, learning rate analyses have been performed on the values retrieved with this procedure. To avoid bias in learning rate comparisons, the same priors were used for all learning rates. In the main analysis, a single set of parameters was used to fit all conditions. In a control analysis, different sets of parameters were used to fit each condition (“Symmetric”, “Asymmetric” and “Reversal”).

### Parameter correlation and parameter recovery

To validate our results, and more specifically to verify that valence-induced differences in learning rates reflected true differences in learning, as opposed to an artefact of the parameter optimisation procedure, we checked the correlations between the free parameters (Experiment 1: β, $\alpha_{c}^{+},\;\alpha_{c}^{-}$; Experiment 2: β, α_CON_, α_DIS_) and the capacity of recovering the correct parameters using simulated datasets. To check the capacity of recovering the correct parameters using simulated datasets, we simulated performance on our behavioural task using virtual participants with parameters values corresponding to those retrieved from our experimental participants [23]. We simulated N = 100 virtual experiments.

## Supporting information

S1 Fig Stability of learning biases across task conditions. (A) Task conditions. The ‘Symmetric’ condition was characterised by a stable reward contingency and no correct option, because the two options had equal reward probabilities. The ‘Asymmetric condition’ was also characterised by a stable reward contingency and a correct option, since one option had a higher reward probability than the other. The ‘Reversal’ condition was characterised by an instable reward contingency: after 12 trials the reward probability reversed across symbols, so that the former correct option became the incorrect one, and vice versa. Note that the number of trials refers to one session and participants performed two sessions, each involving new pairs of stimuli (192 trials in total). (B) and (C) Computational results as a function of the task conditions in Experiment 1 and Experiment 2, respectively. Each column presents the result of the corresponding condition presented in (A).
(EPS)

S2 Fig Parameter recovery in Experiment 1. “True values”: learning rates used to simulate the data. “Recovered values”: learning rates obtained from the simulations once the same parameter optimisation was applied as for the experimental data. “Case: unbiased”: no learning rate bias. “Case: biased”: positivity learning rate bias.
(EPS)

S3 Fig Parameter recovery in Experiment 2. “True values”: learning rates used to simulate the data. “Recovered values”: learning rates obtained from the simulations once the same parameter optimisation was applied as for the experimental data. “Case: unbiased”: no learning rate bias. “Case: semi-biased”: learning rate bias only concerning factual learning. “Case biased”: confirmation bias involving both factual and counterfactual learning.
(EPS)
